# Assessing Impact of Cooking Methods on the Physicochemical and Sensory Properties of Instant Fortified Rice Congee for the Elderly

**DOI:** 10.3390/foods13050723

**Published:** 2024-02-27

**Authors:** Noor Farisya Mohd Shaharom, Anida Yusoff, Siti Roha Ab Mutalib, Eng-Keng Seow

**Affiliations:** 1 Department of Food Science and Technology, School of Industrial Technology, Faculty of Applied Sciences, Universiti Teknologi MARA, Shah Alam 40450, Selangor, Malaysia; 2022963527@student.uitm.edu.my (N.F.M.S.); anida@uitm.edu.my (A.Y.); sitiroha7796@uitm.edu.my (S.R.A.M.); 2 Food Science Research Group, Faculty of Applied Sciences, Universiti Teknologi MARA, Shah Alam 40450, Selangor, Malaysia; 3 Integrative Pharmacogenomics Institute (iPROMISE), Universiti Teknologi MARA, Selangor Branch, Bandar Puncak Alam 42300, Selangor, Malaysia

**Keywords:** instant fortified rice congee, porridge, congee, functional foods, elderly, curcumin, collagen, functional ingredients

## Abstract

Instant rice congee (IRC) fortified with functional ingredients is designed for supplementation in nourishing the elderly. In this study, collagen peptide and curcumin were fortified in IRC to improve antioxidant and protein content. Different cooking methods were used to prepare rice congee in order to retain the nutritional content of instant fortified rice congee (IFRC). The effect of cooking methods on IFRC were investigated in this study using field emission scanning electron microscopy (FESEM) and Fourier transform infrared spectroscopy (FTIR). As for cooking methods, the steaming method (IFRC-S) exhibited the highest total phenolic content (TPC) at 36.13 ± 5.63 mg GAE/g sample; a ferric reducing antioxidant power (FRAP) value of 6.39 ± 0.24 mg TE/g sample and protein content at 52.20 ± 6.48%. There were no significant differences (*p* > 0.05) in the texture analysis of hardness, cohesiveness and viscosity between the different cooking methods. However, the boiling method (IFRC-B) showed the highest adhesiveness, at −58.78 ± 11.55 g/s. IFRC with different cooking methods also had no significant differences (*p* > 0.05) in bulk density, volume expansion and the water absorption index. In sensory analysis, it was found that there were no significant differences (*p* > 0.05) detected in attribute colour, odour, taste, texture and overall acceptability between each cooking method. This study is particularly useful for gaining a preliminary understanding of the development of IRC focused on the elderly.

## 1. Introduction

According to statistics from the World Health Organisation (WHO), the percentage of people over 60 years old in the world will rise from 12 to 22% between 2015 and 2050 [1]. Most countries are seeing an increase in the number and proportion of elderly people in their population. As a consequence, nations worldwide may face tough challenges when it comes to ensuring that their social and health care systems are prepared to sustain an ageing population [2,3]. One of the most important aspects of ensuring elderly health is a balanced diet. When a person ages, their body undergoes significant changes, particularly in the digestive system. For example, they may have difficulty chewing, swallowing and digesting food due to dental issues and gastrointestinal problems [2,3,4,5].

Rice congee is one of the most suitable foods to aid elderly individuals with dental problems in easy chewing and swallowing. Furthermore, rice congee is said to be an ultimate comfort food as it contains high moisture, which helps in digestion and eases inflammation in the stomach [6,7]. Therefore, rice congee is less likely to trigger any discomfort in the stomach, which will lead to diarrhoea. Nevertheless, rice congee is a carbohydrate-based food that has a low protein content and other nutrients. Plain rice congee is usually made using three basic ingredients, which are rice, water and salt. The nutrients supplied by rice congee may not be sufficient for the elderly, and this calls for the need for fortification with functional ingredients. Thus, instant fortified rice congee (IFRC) is designed for supplementation in nourishing elderly people with a consideration of its characteristics, including its extended shelf life, and the fact that it is simple to prepare and relatively easy to manage in the transportation and distribution process [8]. 

Rice congee can be prepared using different methods such as simmering, boiling, steaming and cooking using an electronic rice cooker. However, simmering is a method that is widely used and conventional in countries such as China, Korea, Japan, Thailand, Malaysia and Indonesia. The simmering method is carried out by cooking rice grains with an amount of water that is ten to twenty times that of the rice in a pot with medium heat for 30 min to 1 h [7,8,9,10]. Then, the steaming method is used to prepare rice congee with a different texture in comparison to that under the simmering method. Typically, rice congee that is cooked using the steaming method will not dry out and adhere to the pot, making the rice congee less viscous. Moreover, steaming, also known as the double boiling method, is said to retain nutrients more than the simmering method does as direct heat is not applied to cook the rice congee. Nowadays, the most convenient way to prepare rice congee is cooking using an electronic rice cooker. Every Asian household owns an electronic rice cooker, which makes it more convenient to cook rice congee. An electronic rice cooker is able to reduce the time spent stirring the rice congee in the pot on the stove. Different methods have different cooking temperatures, which means rice congee can have different nutritional contents. 

As stated by Hashim et al. [11], collagen is primarily present in all connective tissues in the human body. However, the production of collagen decreases with age and bad diets. Hence, commercial collagen extracted from marine life such as fish, sponges and jellyfish is fully utilised in the industry. For example, marine-based collagen can be used as a food emulsion to encapsulate fish oil for protection and for masking its fishy smell. Furthermore, marine-based collagen might serve as a potential antioxidant in nutraceutical and pharmaceutical products [12]. In addition, the potential use of marine collagen, particularly in the production of food, has received considerable attention from researchers. This is because an alternative way for humans to obtain collagen is through their diet. Thus, numerous studies have utilised collagen as a functional ingredient to be incorporated into different kinds of foods and drinks. A large number of commercial food products on the market nowadays use collagen, including chocolate drinks with collagen, juice with collagen and bird’s nest drink with collagen. Previous research by Blinnikova et al. [13] led to the production of fruit-jelly sweets enriched with collagen. The technological process of producing sweets meets the requirements of regulatory documents by obtaining a product with high nutritional value that fulfils the required content for biologically active substances and has good organoleptic properties. These fruit-jelly sweets contribute 0.36% of protein out of the recommended daily intake of protein for the elderly. Therefore, collagen could be a good functional ingredient to fortify instant rice congee (IRC) with as a source of protein for the elderly. 

According to Tripathy et al. [14], curcumin is rich in antioxidants, is proven to be capable of improving the immunity of the human body and may fight viral infections. In addition, curcumin is said to have anti-inflammatory, anti-aggregatory, anti-apoptotic, anti-proliferative and anti-cancer properties [15]. Research by Munekata et al. [16] has provided evidence that curcumin has little effect on physicochemical properties, enhances antioxidant capacity and slows microbial growth, but can affect the colour and sensory qualities of food products. Previous research by Adegoke et al. [17] led to the development of a functional biscuit made from wheat, soya bean and turmeric (*Curcuma longa*), mainly curcumin. Biscuits with turmeric powder showed the greatest positive impact on antioxidant properties. Hence, curcumin could be a suitable choice of functional ingredient to fortify instant rice congee with in order to aid the elderly in healthy eating.

Furthermore, incorporating curcumin in amounts exceeding 5% impacts the textural properties of fried rice snacks by increasing hardness and decreasing crispiness [18]. Additionally, fortification with collagen leads to significant improvements in the rheological properties of surimi, sausages and frankfurters [11]. To summarize, the addition of collagen and curcumin influences not only the protein and antioxidant properties but also the textural properties of products. Instant rice congee fortified with collagen and curcumin could be a protein- and antioxidant-rich option, potentially serving as a nutritious food with an appropriate texture that is suitable for the elderly.

## 2. Materials and Methods

### 2.1. Raw Materials

Imported white rice grains (*Oryza sativa* L.), brand Jasmine Super 5, were purchased from supermarkets in Selangor, Malaysia. The white rice grains were placed in a tight container and stored in a dark place prior to analysis. Collagen Fortigel B and curcumin cavacurmin were sponsored by a local ingredient supplier (DKSH, Selangor, Malaysia).

### 2.2. Chemicals and Reagents

The chemicals and reagents that were used in this research were gallic acid 97.5–102.5% (Sigma-Aldrich, Schnelldorf, Germany), ascorbic acid (R&M, Selangor, Malaysia), 2,2-diphenyl-1-picrylhydrazyl (DPPH) (Sigma-Aldrich, Schnelldorf, Germany), sodium carbonate anhydrous (R&M, Selangor, Malaysia), boric acid AR/ACS (R&M, Selangor, Malaysia), Folin–Ciocalteau phenol reagent (R&M, Selangor, Malaysia), iodine 1% solution (R&M, Selangor, Malaysia), quercetin pharmaceutical secondary standard (Supelco, Bellefonte, PA, USA), amylose from potato used as an amylase substrate (Sigma Aldrich, Burlington, MA, USA), 2,4,6-tris(2-pyridyl)-s-triazine (Sigma Aldrich, Burlington, MA, USA), iron (III) chloride hexahydrate (R&M, Selangor, Malaysia), sodium acetate anhydrous (R&M, Selangor, Malaysia), 6-hydroxy-2,5,7,8 tetramethylchromane-2-carboxylic acid (Sigma Aldrich, Burlington, MA, USA), hydrochloric acid (R&M, Selangor, Malaysia), methanol (R&M, Selangor, Malaysia), sodium hydroxide (R&M, Selangor, Malaysia), sulphuric acid (R&M, Selangor, Malaysia) and acetic acid (R&M, Selangor, Malaysia). 

### 2.3. Preparation of Instant Rice Congee (IRC)

The white rice grains were ground using a mini electric grinder with a speed of 3000 rpm. Then, the ground rice was sieved using a 1 mm sieve. The ground rice grains measuring about 1 mm were cooked using three different methods: boiling (IRC-B), steaming (IRC-S) and cooking using an electronic rice cooker (IRC-E). The ratio of rice to water used to cook the rice congee was 1:10, and the heating process was maintained for 15 min at constant temperature, at 100 °C for IRC-B and IRC-E, and at 98–99 °C for IRC-S [10,19,20]. For IFRC, after the temperature dropped to 60 °C for each cooking method, 500 mg of curcumin and 12.5 g of collagen (the recommended daily intakes for curcumin and collagen) were added to 100 g of (semi-solid) cooked rice congee [21,22]. The rice congee was stirred until the collagen and curcumin were mixed completely or the texture was smooth without any lumps (IFRC-B, IFRC-E and IFRC-S). IRC and IFRC cooked using three different methods were spread on a tray for the drying process. Then, the trays were placed in an oven at 60 °C until the moisture content of the dried IRC and IFRC reached 6–7% [23,24]. The dried IRC and IFRC were ground and sieved using a 1 mm sieve [25]. Then, the dried IRC and IFRC were kept in a sealed aluminium bag until further analysis.

### 2.4. Chemical Composition Analysis

#### 2.4.1. Amylose Content Analysis

Samples of IRC and IFRC were analysed for amylose content based on the method explained by Jayaraman et al. [26]. The colorimetric method used is a reaction between amylose and iodine. About 100 mg of defatted samples was mixed with 1 mL of 95% ethanol, and 10 mL of 1 N NaOH was added gently. The mixture was left overnight, and the solution was prepared up to an amount of 100 mL in a volumetric flask. Next, 1 mL of 1 N acetic acid and 2 mL of iodine solution (0.2% I_2_ in 2% KI) were added to approximately 5 mL of the aliquot from the stock solution. Then, a volume of 100 mL of distilled water was added and the solution was mixed thoroughly in a volumetric flask. The solution was left for 20 min at room temperature. The absorbance of the solution was measured at 620 nm using a Genesys 20 spectrophotometer (Thermo Scientific, Waltham, MA, USA). The blank used was 5 mL of 1 N NaOH mixed with 1 mL of 1 N acetic acid and 2 mL of iodine solution, the volume of which then reached 100 mL after adding distilled water. The amylose content in the samples was calculated based on the standard curve (Y = 41.35X + 0.16; R^2^ = 0.98) of potato amylose with a series of concentrations: 0.001%, 0.002%, 0.003%, 0.004% and 0.005%.

#### 2.4.2. Protein Determination

The protein contents of IRC and IFRC were determined using the Kjeldahl method by the AOAC [27]. About 0.6 g of defatted IFRC samples was weighed into the Kjeldahl tube. A catalyst tablet was added followed by the addition of 25 mL of 98% H_2_SO_4_. The sample was heated up to 150 °C for 30 min, then to 300 °C for 60 min and lastly to 420 °C for 5 h for the digestion process. Then, the digestion tube was left to stand to cool for 2 h. Following the digestion process, 2% of H_3_BO_3_ and deionised water were added to the tank of the distillation unit. The digestion tube that contained the sample was attached to the distillation unit, followed by the addition of 32% of NaOH. Then, 0.1 M of HCl was prepared in a Schott bottle for the titration process with 2% of H_3_BO_3_. The distillation process was run automatically followed by a titration process. The percentage of protein content was calculated as described below:

(1) $$\mathrm{Kjeldahl}\;\mathrm{nitrogen},\;\%=\frac{\left[\left(VS-VB\right)\right]\times M\times 14.01}{W\times 10}$$

Crude protein, % = % Kjeldahl nitrogen N × F(2)

where VS = volume (mL) of standardised acid used to titrate a sample; VB = volume (mL) of standardised acid used to titrate the reagent blank; M = molarity of standard HCl; 14.01 = atomic weight of N; *W* = weight (g) of the test portion or standard; 10 = factor to convert mg/g into percent; and F = factor to convert N into protein (5.95).

### 2.5. Determination of Texture Analysis and Viscosity

#### 2.5.1. Rehydration of IRC and IFRC

About 10 g of dried IRC and IFRC samples was rehydrated with 70 mL of boiling water, making the ratio of rice to water 1:7. Then, the mixture was stirred until the texture of the rice congee was smooth without any lumps [24].

#### 2.5.2. Texture Analysis

About 10 g of rehydrated IRC and IFRC (semi-solid) samples with a 23 mm diameter and 35 mm height was placed in a beaker. The rehydrated rice congee was analysed using a TA.XT Plus texture analyser (Stable Micro Systems, Godalming, UK) fitted with a 50 kg load cell and a cylinder-shaped flat probe (P/20) measuring 20 mm in diameter. The probe was working at a speed of 1 mm/s at a distance of 30 mm into each congee sample. The maximum force value of the first peak, the negative area of the graph and the ratio of areas under the two peaks were recorded as hardness (N), adhesiveness (N/s) and cohesiveness values, respectively [28].

#### 2.5.3. Viscosity

The viscosity of the rehydrated IRC and IFRC was determined using a RVT viscometer (Brookfield, Middleboro, MA, USA) based on the method stated by Mahgoub et al. [29] with some modifications. About 80 g of the rehydrated IRC and IFRC was measured using spindle #7 at a shear rate of 50 rpm in 30 s. The results were expressed in centipoise, cP. 

### 2.6. Determination of Antioxidants Capacity of Dried IRC Samples

#### 2.6.1. Sample Preparation

About 0.5 g of ground dried IFRC samples was weighed accurately, extracted with 20 mL of acidified methanol/1 N HCl (85:15 *v*/*v*) in a set of capped centrifuge tubes for 1 h and vortexed for 2 min. Then, the soluble extract was centrifuged at 5000 rpm for 10 min and the supernatant was collected. The residues were mixed with 20 mL of extracting solvent using the same method, and all supernatants were pooled and stored at 4 °C in the dark until further analysis [30]. 

#### 2.6.2. Total Phenolic Content (TPC)

TPC was determined using Folin–Ciocalteau method by Mayachiew et al. [31] with some modifications. About 200 µL of an aliquot from the extracted sample was mixed with 1.5 mL of freshly diluted 10-fold Folin–Ciocalteau reagent, and the mixture was incubated for 5 min. Then, 1.5 mL of 60 g/L sodium carbonate solution was added to the incubated solution. The solution was left at room temperature for 90 min for another round of incubation. The absorbance of the solution was measured at 750 nm using a Genesys 20 spectrophotometer (Thermo Scientific, Waltham, MA, USA). A standard curve (Y = 0.80X + 0.02; R^2^ = 0.99) was prepared by using gallic acid as a standard in a series of concentrations: 0.2 mg/mL, 0.4 mg/mL, 0.6 mg/mL, 0.8 mg/mL and 1.0 mg/mL. The TPC of the extracted dried rice congee was expressed in mg of gallic acid equivalents (GAEs) per g of dry sample (mg GAE/g dry sample). 

#### 2.6.3. Total Flavonoid Content (TFC)

The method explained by Goyat et al. [28] was used to measure the TFC in the prepared extract using the quercetin standard. About 1 mL of an aliquot of the extracted sample was mixed with 0.3 mL of 5% (*w*/*v*) sodium nitrite solution. After 5 min, 0.3 mL of 10% aluminium chloride (AlCl_3_) was added to the solution followed by 2 mL of 1 M sodium hydroxide (NaOH) after 1 min. Then, the mixture solution was diluted with 6.4 mL of distilled water and vortexed for 1 min. The absorbance of the samples was read at 510 nm against distilled water as a blank using a Genesys 20 spectrophotometer (Thermo Scientific, MA, USA). A standard curve (Y = 0.52X + 0.01; R^2^ = 0.99) was prepared using quercetin as a standard in a series of concentrations: 0.1 mg/mL, 0.2 mg/mL, 0.3 mg/mL, 0.4 mg/mL and 0.5 mg/mL. The result was expressed in milligrams of quercetin equivalents (QAE) per gram (mg QAE/g) of sample.

#### 2.6.4. 2,2-Diphenyl-1-picrylhydrazyl (DPPH) Radical Scavenging Activity

The DPPH radical scavenging activity of the sample extract was determined based on the method described by Mayachiew et al. [31] with some modifications. About 1 mL of an aliquot of the sample extract was mixed with 2 mL of 0.5 mM 2,2-diphenyl-1-picrylhydrazyl (DPPH), which had been diluted in methanol. The mixture was incubated for 30 min in the dark at room temperature. The absorbance of the samples was measured at 517 nm using the Genesys 20 spectrophotometer (Thermo Scientific, Waltham, MA, USA). Ascorbic acid was used as a positive control (Y = 0.03X + 0.03). The percentage of scavenging activity was calculated as follows:

(3) $$\%\;\mathrm{scavenging}=\frac{\left(Absorbance\;of\;control\;-\;Absorbance\;of\;test\;sample\right)}{Absorbance\;of\;control}\times 100\%$$

#### 2.6.5. Ferric Reducing Antioxidant Power (FRAP)

The method described by Mayachiew et al. [31] was used to determine the FRAP of extracted samples with some modifications. FRAP reagent was prepared by mixing 10 mM TPTZ solution with 40 mM HCl, 20 mM FeCl_3_·6H_2_O and 300 mM acetate buffer (pH 3.6) at a ratio of 1:1:10 [*v*/*v*/*v*]. Then, 1.5 mL of prepared FRAP reagent was mixed with 50 μL of the extracted sample in an amber test tube and incubated in a water bath at 37 °C for 30 min. The absorbance of the ferrous tripyridyltriazine complex solution, which is a coloured product, was measured at 593 nm using the UV-vis spectrophotometer. FRAP was calculated based on the standard curve (Y = 1.69X + 0.01; R^2^ = 0.98) of Trolox with a series of concentrations: 0.02 mg/mL, 0.04 mg/mL, 0.06 mg/mL, 0.08 mg/mL and 0.10 mg/mL; the results were expressed in milligrams of Trolox equivalents (TE) per gram (mg TE/g) of dry sample.

### 2.7. Physicochemical Analysis 

#### 2.7.1. Volume Expansion

Volume expansion was evaluated in accordance with the method by Prapluettrakul et al. [24] with some modifications. About 20 g of the dried IRC and IFRC samples was placed in a measuring cylinder, and the measuring cylinder was tapped gently on a flat surface until a constant volume was achieved. The volume was recorded. Then, hot water was added and the mixture was left for 10 min. The expanded volume was recorded and calculated as follows:

(4) $$\mathrm{Volume}\;\mathrm{expansion}=\frac{Expanded\;volume\;of\;sample\;(mL)}{Volume\;of\;dried\;sample\;(mL)}$$

#### 2.7.2. Bulk Density

The bulk density of the samples was measured following the method by Mayachiew et al. [31]. About 20 g of dried IRC and IFRC was poured into a 50 mL measuring cylinder. The measuring cylinder was tapped gently on a flat surface until a constant volume was obtained. The bulk density was calculated as the mass of the sample (g) divided by its volume (mL).

#### 2.7.3. Water Absorption Index (WAI)

The WAI was evaluated in accordance with the method of Woraratphoka et al. [32]. Approximately 2.5 g of dried IRC and IFRC was hydrated in the centrifuge tube by adding 30 mL of distilled water. The rice congee mixture was incubated at room temperature for 30 min, while being mixed every 5 min. Then, the sample was subjected to centrifugation at 2200 rpm for 15 min. The supernatant was decanted, and then the residue was weighed. The WAI was determined as follows:

(5) $$\mathrm{WAI}=\frac{Weight\;of\;residue\;(g)}{Weight\;of\;sample\;(g)}$$

#### 2.7.4. Colour Analysis

The colour of the rehydrated IRC and IFRC samples was determined using a CR400 chromameter (Konica Minolta, Tokyo, Japan), which was calibrated with a white calibration tile. The data for white calibration were set at Y = 92.9, x = 0.3114 and c = 0.3190. Three parameters, L* (lightness), a* (redness (+), and greenness (−)), b* (yellowness (+) and blueness (−)) were measured [31]. 

#### 2.7.5. Field Emission Scanning Electron Micrograph (FESEM)

IRC and IFRC powdered samples were spread evenly on a 2 cm circular aluminium stud with double-sided sticky tape and sputter-coated with gold using sputter coater SCD 005 (BAL-TEC, Pfäffikon, Switzerland). Then, the surface morphology of samples was examined and photographed using a field emission scanning electron microscope, (FESEM) Supra 40VP (ZEISS, Oberkochen, Germany), at an accelerating voltage of 5 kV [33]. 

#### 2.7.6. Fourier Transform Infrared Spectroscopy (FTIR)

IRC and IFRC powdered samples were placed in a pellet holder. The samples were analysed using a Spectrum One FTIR spectrometer (Perkin Elmer, Waltham, MA, USA). The spectra were collected at a threshold resolution of 0.5 from 4000 to 650 cm^−1^ at room temperature with 4 scans per recorded spectrum. Then, the peak intensities at 995, 1020 and 1047 cm^−1^ in the deconvoluted spectrum were recorded to represent the degree of amylose inclusion and the degree of the short-range molecular order of C-C and C-O bonds [32]. 

### 2.8. Sensory Evaluation of IFRC 

Sensory evaluation involving panellists at an old folks’ home around Selangor, Malaysia, was carried out. Consent for participation in this study was obtained from each elderly individual. Elderly individuals aged 60 years old and above were screened, and those without diabetes were selected. In total, 50 panellists were enlisted to evaluate the samples using a 9-point hedonic scales to assess the acceptability of each sample from 1 to 9, where 1 = disliked very much; 9 = liked very much. The sensory attributes, which were the colour, odour, taste, texture (stickiness) and overall acceptability of IFRC, were evaluated by the panellists. Each panellist received a tray with samples that were randomly coded with 3-digit numbers, a glass of water and an evaluation form. Panellists were briefed on the guidelines for each attribute to ensure that the panellists evaluated the samples correctly [9]. This study was approved by Research Ethics Committees from the Faculty of Applied Sciences, Universiti Teknologi MARA, Malaysia, with the reference number REC/06/2023(PG/MR/191). 

### 2.9. Statistical Analysis 

All results were expressed in mean ± standard deviation ($\bar{x}$ ± SD). Statistical analysis was performed using SPSS version 28 (IBM Corp., Armonk, NY, USA). One-way analysis of variance (ANOVA) and Tukey’s multiple comparison test were used to identify significant differences. Differences were significant when *p* < 0.05.

## 3. Results and Discussion

### 3.1. Formulation of IRC

This research was based on the formulation of IRC with mixtures of 12.5 g of collagen and 500 mg of curcumin. The recommended daily intake for collagen peptide is between 2.5 and 12.5 g per day in order for it to be functionally effective in the human body. Five grams per day has shown the potential to improve bone density while a daily intake of ten grams of collagen peptide shows an improvement in muscle mass and body composition [22,34]. Furthermore, adults are recommended to take 500 mg of curcumin per day in order for curcumin to be effective as an antioxidant in the human body [21]. The moisture contents of IRC and IFRC prepared using different cooking methods are displayed in Table 1. As stated, there were no significant differences (*p* > 0.05) between each sample. The moisture content of each sample of IRC was less than 7%. This result is in agreement with that of research conducted by Kanhariang et al. [23], where the moisture content of IRC was below 7%.

### 3.2. Chemical Composition Analysis

The amylose and protein contents for each sample of IRC and IFRC are shown in Table 2. IRC-B and IFRC-B show the highest values of amylose content, which are 0.76% and 0.58%, respectively. This might be due to the higher temperature used in cooking IRC-B and IFRC-B as compared to that used for cooking IRC-S. According to Chen et al. [10], amylose leaches out from the granules as they expand and rupture during the cooking process. The amount of amylose leaching is dependent on the cooking temperature. As a results, amylose content increases significantly (*p* < 0.05) in IRC-B and IFRC-B in comparison with that under the other methods. Meanwhile, IFRC-S exhibits the highest protein content retention of 52.20% after the addition of collagen peptide in IRC. Based on Chen et al. [10], protein denaturation gradually increases as temperature and cooking time increase, causing severe damage to the starch–protein framework. Protein content in IRC showed no significant differences (*p* > 0.05). Therefore, the results indicate that following the fortification of collagen, IFRC-S retains the highest protein content, suggesting a relatively lower degree of protein denaturation in the steaming method, possibly due to the lower cooking temperatures used in this process.

### 3.3. Texture and Viscosity of IRC and IFRC

The texture and viscosity of IRC and IFRC for each cooking method are reported in Table 3. The textural parameters determined were hardness, adhesiveness and cohesiveness. Hardness showed significant differences (*p* < 0.05) between IRC and IFRC. IRC has a higher value of hardness as compared to IFRC. The addition of collagen peptide was responsible for the decreased value of hardness. Increased protein in the starch slowed down the process of water absorption and the swelling of starch, making the IFRC softer [35]. This is in agreement with the result reported by Shim and Lim [9], in which mixed-grain porridges with high protein had significantly (*p* < 0.05) lower values of hardness as compared to plain rice congee. The adhesiveness and cohesiveness of IFRC significantly increased (*p* < 0.05) in comparison to those of IRC. Although IRC had a higher amylose content than did IFRC samples cooked using the same methods, the inclusion of collagen peptide in IFRC contributed to its adhesiveness. The rise in cohesiveness and adhesiveness was likely due to the addition of collagen peptide. Liu et al. [36] reported that cohesiveness and adhesiveness increased when rice starch was distributed evenly between the gel networks of collagen peptide. However, there were no significant differences (*p* > 0.05) between each method of cooking IFRC in terms of cohesiveness while IFRC-B showed the highest value of adhesiveness in IFRC. IFRC-B had the highest amylose content, exhibiting high adhesiveness as compared to that under other cooking methods. A more linear amylose chain in starch will promote efficient entanglement and increase hydrogen bonding between adjacent polymeric chains, which enhances adhesiveness [37]. 

Moreover, collagen peptide has a high concentration of protein, which may restrain starch granule hydration and swelling, resulting in decreased viscosity. The decreasing value of viscosity is due to the formation of amylose inclusion complexes between protein and amylose from starch [31]. This result is in agreement with that of a study by Shao et al. [35], which showed that yam powders with the highest protein ratio had the lowest viscosity. 

### 3.4. Antioxidant Capacity of IFRC

In accordance with the findings reported by Mayachiew et al. [31], the TPC of plain rice congee was recorded as 1.09 ± 0.08 mg GAE/g sample, and FRAP was measured to be 2.76 ± 0.04 mg TE/g sample. Additionally, Shim and Lim [9] determined that the DPPH of rice congee is approximately 10%. Based on Table 4, fortification with collagen and curcumin increased the TPC, FRAP and DPPH of rice congee as compared to those of plain rice congee as reported by Mayachiew et al. [31], and Shim and Lim [9].

As shown in Table 4, there were no significant differences (*p* > 0.05) detected for TFC, which was in the range of 5–6 mg of quercetin equivalents per gram of sample of IFRC. Furthermore, DPPH radical scavenging activity falls within the range of 14–15% for IFRC samples. Curcumin is responsible for the higher value of DPPH radical scavenging activity. A similar result was reported by Thuy et al. [38], where a higher value of DPPH radical scavenging activity was detected (about 27.25%) in waffles fortified with curcumin, lecithin and canola oil as compared to that of a control sample.

IFRC-S shows the highest values for TPC and FRAP, which are 36.13 ± 5.63 mg GAE/g sample and 6.39 ± 0.24 mg TE/g sample, respectively. IFRC-S has the lowest cooking temperature, at about 98–99 °C, as compared to that of the other two cooking methods, which is 100 °C. This result agrees with that of research conducted by Renzo et al. [39], which found that samples of turmeric cookies baked at a higher temperature (180 °C) had lower antioxidant activity in comparison to samples of turmeric cookies baked at 150 °C. Most of the antioxidants such as polyphenol can be made thermolabile via heat treatment, which results in antioxidant degradation. 

### 3.5. Physicochemical Properties

#### 3.5.1. Volume Expansion, Bulk Density and WAI

The IRC and IFRC samples were analysed for volume expansion, bulk density and the WAI. The volume expansion of IRC was significantly (*p* < 0.05) higher than that of IFRC. This might have been due to the fortification with curcumin in IFRC. Curcumin is a hydrophobic phenolic compound that limits the volume expansion of IFRC [14]. As shown in Table 5, IRC samples have a higher value for bulk density as compared to the IFRC sample even though there are no significant differences (*p* > 0.05) between the samples. This result is similar to that of the study reported by Mayachiew et al. [31], where the highest bulk density was reported in the control rice porridge while the addition of soybean and mung bean decreased the bulk density of the instant rice porridge. This might have been due to the gelatinisation process being slowed down by the addition of protein from functional ingredients. Moreover, Thomas et al. [40] mentioned that bulk density corresponds with the water uptake ratio. Thus, a high bulk density will give a high water uptake ratio. Then, IRC has a significantly (*p* < 0.05) higher value for the water absorption index (WAI) as compared to IFRC samples. This might be due to the addition of collagen peptide and curcumin, which lowers the value of the WAI. This result is in agreement with that of Kanhariang et al. [23], where the addition of egg white reduced the value of the WAI. Moreover, Khuenpet et al. [41] also reported that the addition of dietary fibre from Jerusalem artichoke to instant congee also decreased the value of the WAI. There are no significant differences (*p* > 0.05) between each cooking method for the WAI of both IRC and IFRC. 

#### 3.5.2. Colour of IRC and IFRC

Based on Table 6, colour analysis for IRC and IFRC showed that L* (brightness) values decreased in IFRC samples as compared to IRC samples, which resulted in a darker colour of IFRC. Curcumin has a predominant yellow colour, so the addition of curcumin significantly (*p* < 0.05) affected the colour of IRC and resulted in the decrease in the L* value of IFRC [18]. This result is supported by Adegoke et al. [17], who reported that biscuits formulated with the flour blends containing turmeric powder had lower L* values than did the control, and thus resulted in a darker colour. Furthermore, a positive a* value indicates redness while a negative a* value shows the greenness of a sample, whereas a positive b* value measures yellowness and a negative b* value indicates the blueness of a sample. Fortification with curcumin also lead to a higher value of a* and b* in IFRC as compared to those in IRC. This result is in agreement with that of with Lim and Han [18], who showed that a fried rice snack fortified with turmeric powder had a dramatically increased a* and b* value. Furthermore, IFRC-S exhibits a lower L* value and higher a* and b* values as compared to those of samples prepared using other methods. This could be attributed to the higher retention of curcumin, aligning with its high antioxidant capacity, as indicated in Table 4. The use of a low cooking temperature in preparing IFRC-S may contribute to the retention of a higher amount of curcumin as compared to that under other methods. The brightness (L* value) decreases as curcumin increases while a* and b* values increase as curcumin increases [17,18]. 

#### 3.5.3. Field Emission Scanning Electron Micrograph (FESEM)

**Figure 1:**
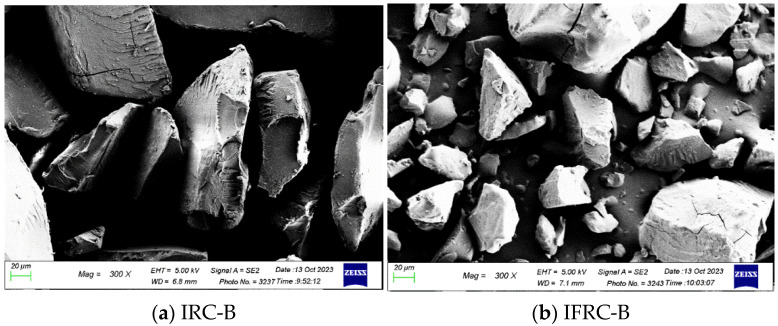

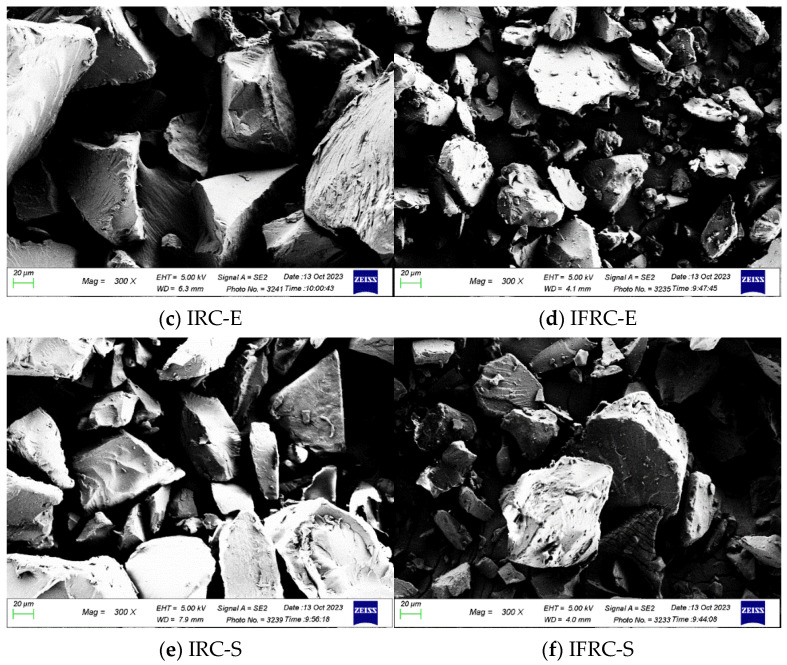
Scanning electron microscopy images of IRC and IFRC. (**a**) IRC cooked using boiling method; (**b**) IFRC cooked using boiling method; (**c**) IRC cooked using electronic rice cooker; (**d**) IFRC cooked using electronic rice cooker; (**e**) IRC cooked using steaming method; (**f**) IFRC cooked using steaming method.

Figure 1 depicts the results of the surface morphology, size and physical form of IRC and IFRC as analysed using FESEM. Each sample had an irregular shape with a compact physical form. However, IRC under each cooking method had a smooth surface morphology, while IFRC samples had small particles attached on the surface of the rice particles. This might have been due to the fortification of IRFC with curcumin and collagen peptide. This result is in agreement with that of Herminiati et al. [42], who reported that the surface of instant congee became denser when inulin was used to fortify instant congee. Moreover, added functional ingredients in IRC will interact with starch and affect the rehydration process and texture of IFRC [43]. As can be seen in Figure 1f, the surface of IFRC-S has a lot of small particles attached to it in comparison to rice congee prepared using other cooking methods. This could have been due to the high retention of curcumin and collagen peptide in IFRC-S. This is can be seen in the data in Table 2 and Table 4 showing that IFRC-S had significantly (*p* < 0.05) higher protein and antioxidant capacity (TPC and FRAP).

#### 3.5.4. Fourier Transform Infrared Spectroscopy (FTIR)

**Figure 2:**
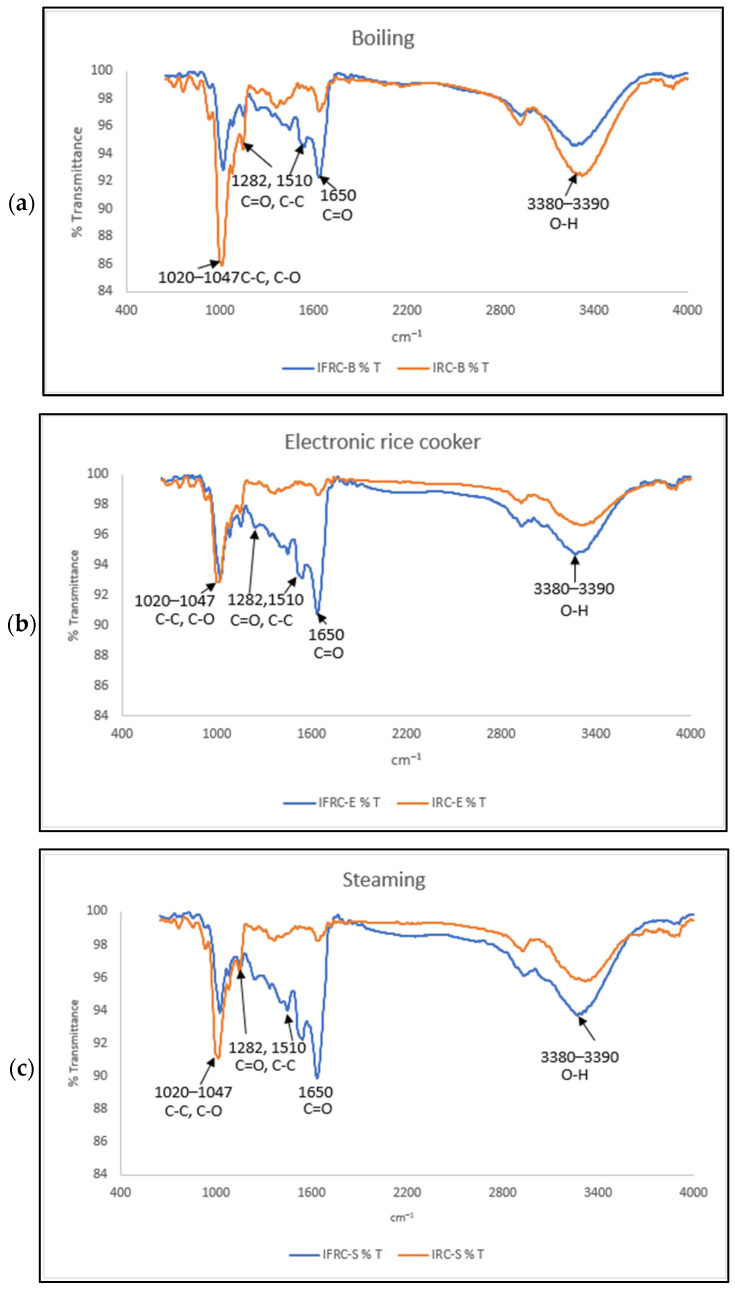
FTIR spectroscopy of IRC and IFRC cooked by (**a**) boiling, (**b**) using an electronic rice cooker and (**c**) via the steaming method.

**Figure 3:**
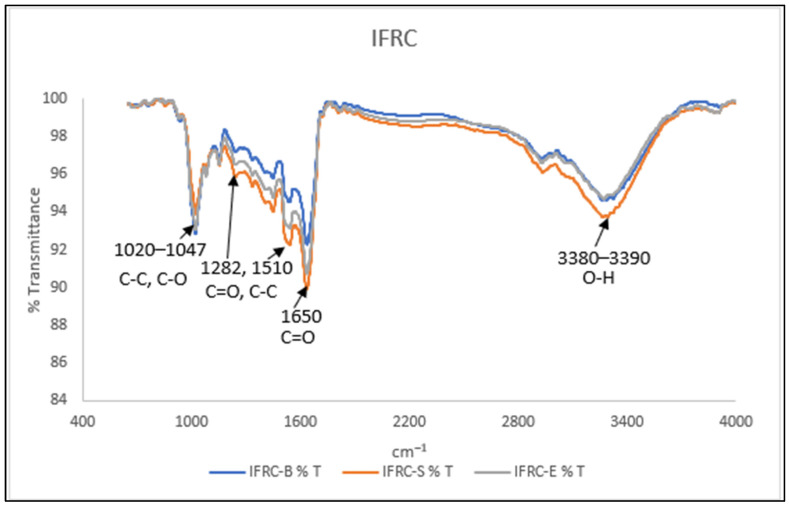
FTIR spectroscopy of IFRC prepared with different cooking methods.

**Figure 4:**
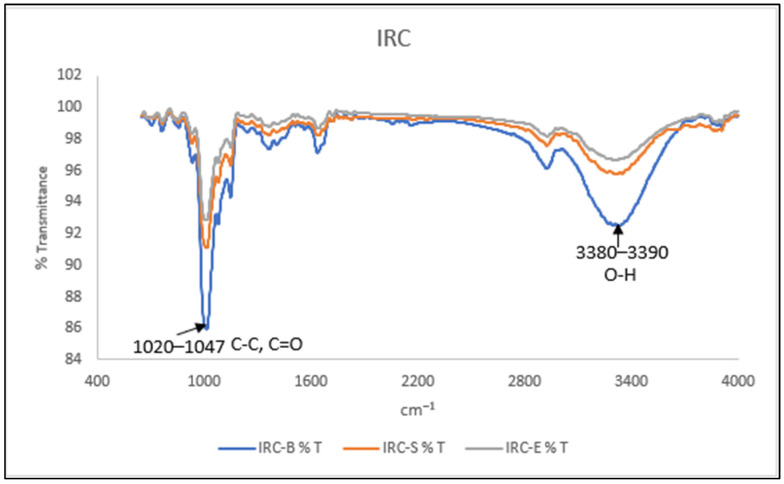
FTIR spectroscopy of IRC prepared with different cooking methods.

Both IRC and IFRC samples were analysed using FTIR to determine their chemical structures. A comparison between IRC and IFRC prepared using an electronic rice cooker, and via boiling and steaming is illustrated via Figure 2a, Figure 2b and Figure 2c, respectively. Fortification with collagen peptide and curcumin was detected between region 1200 and 1650 cm^−1^. FTIR spectra of IFRC and IRC are presented in Figure 3 and Figure 4, respectively. The stretching absorption of FTIR spectra at region 1020–1047 cm^−1^ indicates the characteristic stretching of amylose in the sample approximately located at 995, 1080, and 1150 cm^−1^ for C-C and C-O bonds [32,44]. IRC-B and IFRC-B showed the highest stretching of amylose, similarly to the results shown in Table 2. In addition, a sharp absorption band was detected at 1282 and 1510 cm^−1^, which indicates C=O and C-C from fortification with curcumin [45]. IFRC-S (orange curve) presented the highest retention of curcumin. Collagen peptide-fortified IRC exhibited strong stretching absorption in region 1650 cm^−1^ as compared to IRC alone. Amide I, which is mostly the peptide bond of C=O stretching, is responsible for the band in region 1650 cm^−1^ [46,47]. As stated in Figure 3, IFRC-S shows the highest stretching for protein in comparison to rice congee prepare with the other two cooking methods. Moreover, there is a stretching bond detected in region 3380–3390 cm^−1^, which shows the presence of O-H bonding for both IRC and IFRC. These results agree with those of Thakur et al. [44], who reported that a higher intensity in this region indicates the presence of water in the samples. 

### 3.6. Sensory Analysis

A sensory evaluation of IFRC prepared using different cooking methods involving 50 panellists was performed. Sensory attributes of IFRC prepared using three different methods based on a hedonic scale of 1–9, from disliked very much to liked very much are reported in Table 7. In general, there was no significant difference (*p* > 0.05) in any of the sensory attributes among the IFRC samples prepared with different cooking methods. This suggests that assessing sensory intensity attributes was challenging for panellists. There was a narrow range for every attribute: colour, odour, taste, texture and overall acceptability. The ratings varied for colour attributes between 6.36 and 6.70. Based on this result, IRC-E has the most attractive colour. As shown in Table 6, IFRC-E has a bright colour with the highest yellowness (b* value). It can be concluded that panellists preferred IFRC with a bright yellow colour. IFRC-B has a score of 6.26 ± 1.60 for texture and 6.56 ± 1.50 for overall acceptability. According to the texture analysis, the hardness for IFRC-B is in between that of the other two methods while IFRC-B has the highest value of adhesiveness and cohesiveness. Samples that received sensory scores surpassing a threshold of six were categorised as sensorially acceptable [28]. According to Shim and Lim [9], a score of five is regarded as the limit of acceptability on a nine-point hedonic scale. 

## 4. Conclusions

Findings in the present study showed that instant fortified rice congee has significantly (*p* < 0.05) decreased viscosity and hardness, and increased adhesiveness and cohesiveness as compared to instant rice congee. Instant fortified rice congee samples showed a significantly (*p* < 0.05) lower values for bulk density, volume expansion and the water absorption index compared to those of instant rice congee samples. In addition, instant fortified rice congee had significantly (*p* < 0.05) lower brightness but a higher value in redness and yellowness in colour analysis as compared to instant rice congee samples. The effect of different cooking methods was determined by showing that IFRC-S retained the highest antioxidant capacity and protein content. IFRC-S has a significantly (*p* < 0.05) higher value in total phenolic content, ferric reducing antioxidant power and protein content while IFRC-B and IRC-B exhibits the highest amylose content. These results were supported by field emission scanning electron microscopy, which depicted that the surface morphology of IFRC-S was denser in comparison with that of IFRC-B and IFRC-E. In addition, curcumin and collagen peptide were detected, and IFRC-S had the highest stretching band of curcumin and collagen peptide, as determined via Fourier transform infrared analysis. Moreover, it can be concluded that steaming is the best method with which to prepare instant fortified rice congee due to its high retention of antioxidant and protein content. For sensory analysis, which was evaluated by the elderly, there is no significant difference (*p* > 0.05) in any of the sensory attributes among the samples of instant fortified rice congee prepared with different cooking methods. A study on different drying methods and rehydration ratios should be conducted in the future to provide a suitable texture of instant fortified rice congee that focuses on the elderly. 

## Figures and Tables

**Table 1:** Moisture content of instant rice congee (IRC) and instant fortified rice congee (IFRC).

Sample	Cooking Methods	Moisture Content (%)
IRC	Boiling (IRC-B)	6.31 ± 0.20 ^a^
	Electronic rice cooker (IRC-E)	6.30 ± 0.18 ^a^
	Steaming (IRC-S)	6.31 ± 0.01 ^a^
IFRC	Boiling (IFRC-B)	6.27 ± 0.12 ^a^
	Electronic rice cooker (IFRC-E)	6.30 ± 0.11 ^a^
	Steaming (IFRC-S)	6.30 ± 0.16 ^a^

Values are expressed in mean ± standard deviation (*n* = 3). Means within the same column followed by the same superscript lowercase letters indicate no significant differences (*p* > 0.05) according to Tukey’s multiple comparison test.

**Table 2:** Chemical composition of IRC and IFRC.

Sample	Amylose Content (%)	Protein Content (%)
IRC-B	0.76 ± 0.01 ^a^	3.04 ± 0.55 ^c^
IRC-E	0.50 ± 0.01 ^c^	3.13 ± 0.57 ^c^
IRC-S	0.58 ± 0.02 ^b^	3.40 ± 0.30 ^c^
IFRC-B	0.58 ± 0.05 ^b^	29.45 ± 2.77 ^b^
IFRC-E	0.44 ± 0.03 ^d^	31.25 ± 1.23 ^b^
IFRC-S	0.42 ± 0.02 ^d^	52.20 ± 6.48 ^a^

Values are expressed in mean ± standard deviation (*n* = 3). Means within the same column followed by different superscript lowercase letters indicate significant differences at *p* < 0.05 according to Tukey’s multiple comparison test.

**Table 3:** Texture analysis and viscosity of IRC and IFRC.

Sample	Texture Analysis			Viscosity (cP)
	Hardness (N)	Adhesiveness (N/s)	Cohesiveness	
IRC-B	0.66 ± 0.02 ^a^	−0.33 ± 0.01 ^c^	0.55 ± 0.03 ^b^	5433 ± 58 ^a^
IRC-E	0.67 ± 0.01 ^a^	−0.34 ± 0.01 ^c^	0.53 ± 0.02 ^b^	1333 ± 58 ^c^
IRC-S	0.63 ± 0.03 ^a^	−0.32 ± 0.01 ^c^	0.57 ± 0.02 ^b^	4667 ± 58 ^b^
IFRC-B	0.55 ± 0.03 ^b^	−0.58 ± 0.11 ^a^	0.80 ± 0.06 ^a^	1211 ± 78 ^cd^
IFRC-E	0.58 ± 0.04 ^b^	−0.49 ± 0.03 ^b^	0.79 ± 0.07 ^a^	1144 ± 53 ^d^
IRFC-S	0.55 ± 0.02 ^b^	−0.47 ± 0.03 ^b^	0.76 ± 0.05 ^a^	1088 ± 60 ^d^

Values are expressed in mean ± standard deviation (*n* = 3). Means within the same column followed by different superscript lowercase letters indicate significant differences at *p* < 0.05 according to Tukey’s multiple comparisons test.

**Table 4:** Antioxidants capacity of IFRC.

Sample	Antioxidants Capacity			
	FRAP (mg TE/g Sample)	TPC (mg GAE/g Sample)	TFC (mg QAE/g Sample)	DPPH (%)
IFRC-B	5.94 ± 0.24 ^b^	29.19 ± 0.36 ^b^	5.56 ± 0.80 ^a^	14.23 ± 3.04 ^a^
IFRC-E	5.91 ± 0.27 ^b^	30.03 ± 3.18 ^b^	5.39 ± 0.78 ^a^	14.75 ± 1.42 ^a^
IFRC-S	6.39 ± 0.24 ^a^	36.13 ± 5.63 ^a^	6.17 ± 1.38 ^a^	14.56 ± 3.30 ^a^

Values are expressed in mean ± standard deviation (*n* = 3). Means within the same column followed by different superscript lowercase letters indicate significant differences at *p* < 0.05 according to Tukey’s multiple comparisons test.

**Table 5:** Volume expansion, bulk density and WAI of IRC and IFRC.

Sample	Volume Expansion	Bulk Density	Water Absorption Index (WAI)
IRC-B	6.84 ± 0.36 ^a^	0.88 ± 0.07 ^a^	5.70 ± 0.19 ^a^
IRC-E	6.52 ± 0.94 ^ab^	0.81 ± 0.14 ^ab^	6.05 ± 0.03 ^a^
IRC-S	5.64 ± 0.50 ^b^	0.78 ± 0.08 ^ab^	5.57 ± 0.14 ^a^
IFRC-B	3.35 ± 0.43 ^c^	0.72 ± 0.03 ^b^	3.51 ± 0.76 ^b^
IFRC-E	3.33 ± 0.42 ^c^	0.72 ± 0.02 ^b^	3.55 ± 0.27 ^b^
IFRC-S	3.38 ± 0.32 ^c^	0.75 ± 0.05 ^b^	3.44 ± 0.30 ^b^

Values are expressed in mean ± standard deviation (*n* = 3). Means within the same column followed by different superscript lowercase letters indicate significant differences at *p* < 0.05 according to Tukey’s multiple comparisons test.

**Table 6:** Colour values of IRC and IFRC.

Sample	Colour Values		
	L*	a*	b*
IRC-B	58.74 ± 0.02 ^a^	−1.61 ± 0.05 ^e^	−1.15 ± 0.03 ^e^
IRC-E	55.21 ± 0.05 ^c^	−1.52 ± 0.01 ^d^	−0.42 ± 0.01 ^c^
IRC-S	58.21 ± 0.03 ^b^	−1.62 ± 0.03 ^e^	−0.84 ± 0.03 ^d^
IFRC-B	45.46 ± 0.54 ^d^	4.44 ± 0.56 ^c^	40.77 ± 3.28 ^b^
IFRC-E	45.42 ± 0.28 ^d^	6.23 ± 0.79 ^b^	44.13 ± 2.33 ^a^
IFRC-S	42.85 ± 1.16 ^e^	7.00 ± 0.76 ^a^	42.11 ± 2.05 ^ab^

Values are expressed in mean ± standard deviation (*n* = 3). Means within the same column followed by different superscript lowercase letters indicate significant differences at *p* < 0.05 according to Tukey’s multiple comparisons test.

**Table 7:** Sensory analysis of IFRC cooked using different methods.

Attributes	Colour	Odour	Taste	Texture	Overall Acceptability
IFRC-B	6.62 ± 1.81 ^a^	6.04 ± 1.76 ^a^	5.80 ± 1.97 ^a^	6.26 ± 1.60 ^a^	6.56 ± 1.50 ^a^
IFRC-E	6.70 ± 1.56 ^a^	5.86 ± 1.67 ^a^	5.60 ± 1.85 ^a^	6.08 ± 1.75 ^a^	6.24 ± 1.76 ^a^
IFRC-S	6.36 ± 1.95 ^a^	5.98 ± 1.73 ^a^	5.82 ± 1.73 ^a^	6.20 ± 1.71 ^a^	6.22 ± 1.75 ^a^

Values are expressed in mean ± standard deviation (*n* = 50). Means within the same column followed by the same superscript lowercase letters indicate no significant differences (*p* > 0.05) according to Tukey’s multiple comparisons test.

## Data Availability

The original contributions presented in the study are included in the article material, further inquiries can be directed to the corresponding author.

## References

[1] World Health Organization Ageing and Health Report. October 2022. https://www.who.int/news-room/fact-sheets/detail/ageing-and-health.

[2] Nitikornwarakul C., Wangpradid R., Sirimuangmoon C., Lekjing S., Nishioka A., Koda T. (2022). Nutritious elderly diet: Pigmented rice-porridge from shear-heat milling process. Ital. J. Food Sci..

[3] Ahmad M.H., Salleh R., Man C.S., Pardi M., Rahim N.C.A., Shahril N., Mutalib M.H.A., Shahar S., Ahmad N.A. (2021). Malnutrition among the Elderly in Malaysia and Its Associated Factors: Findings from the National Health and Morbidity Survey 2018. J. Nutr. Metab..

[4] Norman K., Haß U., Pirlich M. (2021). Malnutrition in Older Adults—Recent Advances and Remaining Challenges. Nutrients.

[5] Kiesswetter E., Colombo M.G., Meisinger C., Peters A., Thorand B., Holle R., Ladwig K.-H., Schulz H., Grill E., Diekmann R. (2019). Malnutrition and related risk factors in older adults from different health-care settings: An *enable* study. Public Health Nutr..

[6] Rhim J.-W., Koh S., Kim J.-M. (2011). Effect of freezing temperature on rehydration and water vapor adsorption characteristics of freeze-dried rice porridge. J. Food Eng..

[7] Karen C., Elaine K., Esther T., Faith N., Wai Y.-X., Yan S.-L., Chriselle K., Cristiane H. (2021). Rice Porridge around the World with IDDSI.

[8] Herminiati A., Nurfajrina L.P., Achyadi N.S., Agustina M. (2021). The estimation of shelf life of instant porridge in the different packaging with method of accelerated shelf life testing of arrhenius model. IOP Conf. Ser. Earth Environ. Sci..

[9] Shim S.-M., Lim S.-Y. (2013). Texture properties and radical scavenging ability of porridge products based on beans, grains, and nuts. J. Korean Soc. Appl. Biol. Chem..

[10] Chen C., Jiang S., Li M., Li Y., Li H., Zhao F., Pang Z., Liu X. (2021). Effect of high temperature cooking on the quality of rice porridge. Int. J. Agric. Biol. Eng..

[11] Hashim P., Mohd Ridzwan M.S., Bakar J., Mat Hashim D. (2015). Collagen in food and beverage industries. Int. Food Res. J..

[12] Xu N., Peng X.-L., Li H.-R., Liu J.-X., Cheng J.-S., Qi X.-Y., Ye S.-J., Gong H.-L., Zhao X.-H., Yu J. (2021). Marine-Derived Collagen as Biomaterials for Human Health. Front. Nutr..

[13] Blinnikova O.M., A Babushkin V., Akindinov V.V., Perfilova O.V., Novikova I.M. (2020). Production technology and mathematical method for modeling the formulation of fruit and jelly candies enriched with collagen. IOP Conf. Ser. Mater. Sci. Eng..

[14] Tripathy S., Verma D.K., Thakur M., Patel A.R., Srivastav P.P., Singh S., Gupta A.K., Chávez-González M.L., Aguilar C.N., Chakravorty N. (2021). Curcumin Extraction, Isolation, Quantification and Its Application in Functional Foods: A Review With a Focus on Immune Enhancement Activities and COVID-19. Front. Nutr..

[15] Ferguson J.J.A., Wolska A., Remaley A.T., Stojanovski E., MacDonald-Wicks L., Garg M.L. (2019). Bread enriched with phytosterols with or without curcumin modulates lipoprotein profiles in hypercholesterolaemic individuals. A randomised controlled trial. Food Funct..

[16] Munekata P.E., Pateiro M., Zhang W., Dominguez R., Xing L., Fierro E.M., Lorenzo J.M. (2021). Health benefits, extraction and development of functional foods with curcuminoids. J. Funct. Foods.

[17] Adegoke G.O., Oyekunle A.O., Afolabi M.O. (2017). Functional Biscuits from Wheat, Soya Bean and Turmeric (Curcuma Longa): Optimization of Ingredients Levels Using Response Surface Methodology. Res. J. Food Nutr..

[18] Lim S.-T., Han J.-A. (2016). Improvement in antioxidant functionality and shelf life of yukwa (fried rice snack) by turmeric (*Curcuma longa* L.) powder addition. Food Chem..

[19] Sieti N., Rivera X.C.S., Stamford L., Azapagic A. (2018). Environmental impacts of baby food: Ready-made porridge products. J. Clean. Prod..

[20] Nguyen D.H.D., Tran P.L., Li D., Han J.-A., Hwang J.-Y., Hong W.-S., Lee J.-S., Kim Y.-R., Yoo S.-H., Park J.-T. (2014). Modification of rice grain starch for lump-free cooked rice using thermostable disproportionating enzymes. Food Res. Int..

[21] Hewlings S.J., Kalman D.S. (2017). Curcumin: A Review of Its Effects on Human Health. Foods.

[22] Khatri M., Naughton R.J., Clifford T., Harper L.D., Corr L. (2021). The effects of collagen peptide supplementation on body composition, collagen synthesis, and recovery from joint injury and exercise: A systematic review. Amino Acids.

[23] Kanhariang K., Thongsook T., Chittrakorn S., Jiamyangyuen S. (2023). Formulation Development of Instant Rice Berry Porridge Fortified with Egg White Protein. J. Agric. Food.

[24] Prapluettrakul B., Tungtrakul P., Panyachan S., Limsuwan T. (2012). Development of Instant Rice for Young Children. Sci. Tech. J..

[25] Kim H.R., Kim M.J., Yang Y.H., Lee K.J., Kim M.R. (2010). Effect of Grain Size on the Physicochemical & Nutritional Properties of Beef Porridge. J. Korean Soc. Food Cult..

[26] Jayaraman R., Uluvar H., Khanum F., Singh V. (2019). Influence of parboiling of red paddy varieties by simple hot soaking on physical, nutrient, phytochemical, antioxidant properties of their dehusked rice and their mineral, starch, and antioxidant’s bioaccessibility studies. J. Food Biochem..

[27] AOAC (2002). Association of Official Agricultural Chemists.

[28] Goyat J., Rudra S.G., Suri S., Passi S.J., Dutta H. (2019). Nutritional, Functional and Sensory Properties of Ready-To-Eat Chia and Quinoa Mix Enriched Low Amylose Rice Based Porridge Mixes. Curr. Res. Nutr. Food Sci. J..

[29] Mahgoub S.A., Mohammed A.T., Mobarak E.-A. (2020). Physiochemical, Nutritional and Technological Properties of Instant Porridge Supplemented with Mung Bean. Food Nutr. Sci..

[30] Piyarach K., Sutham P., Pongsiri K.-I., Randah A., Teerawan S. (2021). Production and Quality Evaluation of Young Thai Jasmine Rice Flake Using Drum Dryer. E3S Web Conf..

[31] Mayachiew P., Charunuch C., Devahastin S. (2015). Physicochemical and Thermal Properties of Extruded Instant Functional Rice Porridge Powder as Affected by the Addition of Soybean or Mung Bean. J. Food Sci..

[32] Woraratphoka J., Khongla C., Musika S., Ausavasukhi A., Pianpumepong P., Boonmasongsung D.T., Kiatponglarp W. (2021). Effect of pullulanase debranching on the physical and chemical properties of instant jasmine rice porridges. Songklanakarin J. Sci. Technol..

[33] Loubes M.A., González L.C., Tolaba M.P. (2018). Pasting behaviour of high impact ball milled rice flours and its correlation with the starch structure. J. Food Sci. Technol..

[34] Gonçalves F. (2017). Impact of collagen hydrolysate in middle-aged athletes with knee and ankle osteochondral lesions: A case series. Int. J. Case Rep. Images.

[35] Shao Y., Jiao R., Wu Y., Xu F., Li Y., Jiang Q., Zhang L., Mao L. (2023). Physicochemical and functional properties of the protein–starch interaction in Chinese yam. Food Sci. Nutr..

[36] Liu Y., Tan Z., Huang Y., Liu J., Xu X., Zhu B., Dong X. (2023). pH-shift strategy improving the thermal stability and oxidation stability of rice starch/casein-based high internal phase emulsions for the application in fish cake. Food Chem. X.

[37] Hilmi B., Hamid Z.A.A., Noor S.N.F.M. The effect of amylose content and starch concentration on mechanical properties of eco-friendly denture adhesives (EFDAs). Proceedings of the 3rd International Postgraduate Conference on Materials, Minerals & Polymer (MAMIP) 2019.

[38] Thuy N.M., Van Hao H., Thu L.C.M., Giau T.N., Tien V.Q., Van Tai N., Van Thanh N. (2023). Effect of turmeric starch, lecithin, and canola oil supplements on waffles quality. Food Sci. Technol..

[39] Renzo A., Tedjakusuma F., Surya R. (2023). Cookies Product Development with the Addition of Turmeric Extract Cookies Product Development with the Addition of Turmeric Extract. IOP Conf. Ser. Earth Environ. Sci..

[40] Thomas R., Wan-Nadiah W.A., Bhat R. (2013). Physiochemical properties, proximate composition, and cooking qualities of locally grown and imported rice varieties marketed in Penang, Malaysia. Int. Food Res. J..

[41] Khuenpet K., Polpued R., Leevanichayakul K., Kaveekiew S. (2020). Effect of Different Rice Varieties and Drying Methods on the Quality of Instant Riceberry Porridge Fortified with Jerusalem Artichoke. Thai Sci. Technol. J..

[42] Herminiati A., Kristanti D., Rimbawan R., Astuti I.D., Achyadi N.S., Yuliantika N. (2020). Characteristics of Inulin-Enriched Instant Porridge and its Effectiveness to Increase Calcium Absorption in Infant Rat Models. Curr. Res. Nutr. Food Sci. J..

[43] Fu T., Niu L., Wu L., Xiao J. (2021). The improved rehydration property, flavor characteristics and nutritional quality of freeze-dried instant rice supplemented with tea powder products. LWT.

[44] Thakur A., Vaidya D., Kaushal M., Verma A., Gupta A. (2020). Mineral composition physicochemical properties, FTIR spectra and scanning electron microscopy of rice flour. J. Vitam. Miner..

[45] Wu L., Tian J., Ye X., Fang H., Zhang Z., Xu C., Zhang H. (2020). Encapsulation and Release of Curcumin with the Mixture of Porous Rice Starch and Xanthan Gum. Starch-Stärke.

[46] Ying D., Hlaing M.M., Lerisson J., Pitts K., Cheng L., Sanguansri L., Augustin M.A. (2017). Physical properties and FTIR analysis of rice-oat flour and maize-oat flour based extruded food products containing olive pomace. Food Res. Int..

[47] Bryan M.A., Brauner J.W., Anderle G., Flach C.R., Brodsky B., Mendelsohn R. (2007). FTIR Studies of Collagen Model Peptides: Complementary Experimental and Simulation Approaches to Conformation and Unfolding. J. Am. Chem. Soc..

